# Gender-specific association between the cytoplasmic poly(A) binding protein 4 rs4660293 single nucleotide polymorphism and serum lipid levels

**DOI:** 10.3892/mmr.2015.3823

**Published:** 2015-05-22

**Authors:** JIAN WU, RUI-XING YIN, TAO GUO, QUAN-ZHEN LIN, SHAO-WEN SHEN, JIA-QI SUN, GUANG-YUAN SHI, JIN-ZHEN WU, DE-ZHAI YANG, WEI-XIONG LIN

**Affiliations:** 1 Department of Cardiology, Institute of Cardiovascular Diseases, The First Affiliated Hospital, Guangxi Medical University, Nanning, Guangxi 530021, P.R. China; 2 Department of Molecular Biology, Medical Scientific Research Center, Guangxi Medical University, Nanning, Guangxi 530021, P.R. China

**Keywords:** lipids, apolipoproteins, cytoplasmic poly(A) binding protein 4, single nucleotide polymorphism, environmental factors, coronary heart disease

## Abstract

Cytoplasmic poly(A) binding protein 4 (PABPC4) is an RNA-processing protein which has an important role in regulating gene expression. The association of the *PABPC4* rs4660293 single nucleotide polymorphism (SNP) and serum lipid profiles has, to the best of our knowledge, not previously been studied in the Chinese population. The present study aimed to investigate the association between the *PABPC4* rs4660293 SNP and several environmental factors with serum lipid levels in the Mulao and Han populations. A total of 727 individuals of Mulao nationality and 729 individuals of Han nationality were randomly selected from stratified randomized samples from a previous study by our group. Genotypes of the *PABPC4* rs4660293 SNP were determined via polymerase chain reaction and restriction fragment length polymorphism analyses and subsequently confirmed by direct sequencing. Serum levels of low-density lipoprotein cholesterol (LDL-C) and apolipoprotein (Apo) B were higher in the Mulao group than those in the Han group (P<0.01 for each). The genotypic and allelic frequencies of the *PABPC4* rs4660293 SNP were significantly different between males and females in the Mulao population (P<0.05 for each), while no significant difference was detected between those of males and females amongst the Han population. The frequency of the G allele was higher in Mulao males than in Mulao females (22.12 vs. 13.44%). The G allele carriers were found to have higher total cholesterol (TC), high-density lipoprotein cholesterol (HDL-C) and ApoAI levels in Han females but not in Han males, and lower TC and HDL-C levels in Mulao females but not in Mulao males than those of the G allele non-carriers (P<0.05 for all). These associations were confirmed by multiple linear regression analysis (P<0.05–0.001). Serum lipid parameters were also correlated with multiple environmental factors (P<0.05–0.001). The *PABPC4* rs4660293 SNP was associated with serum TC, HDL-C, LDL-C and ApoAI levels in these study populations; however, the association varied between the Mulao and Han populations. A gender-specific association was identified in the populations of the two ethnic groups.

## Introduction

Coronary heart disease (CHD) remains the leading cause of morbidity and mortality amongst males and females in western society and is of increasing concern in developing countries, despite significant advances in elucidating its underlying pathophysiology (1–5). It was estimated that there were 7.4 million mortalities worldwide from ischemic heart disease in 2012, which accounted for 13.2% of total mortality (6). Furthermore, it has been predicted that CHD will remain the leading cause of mortality until at least 2020 (7). There are numerous CHD risk factors, of which metabolic abnormalities in blood lipids, in particular low-density lipoprotein cholesterol (LDL-C) elevation and high-density lipoprotein cholesterol (HDL-C) depression, are mainly involved in the development and progression of CHD (8) and therefore represent a crucial target for effective therapeutic intervention (9,10). In epidemiological studies, genetics interacting with environmental factors, including diet, nutritional status and physiological parameters, have been shown to exert a significant effect on the dysregulation of lipid metabolism (11–14).

Studies to elucidate the genetic contributions underlying variations in plasma lipid and lipoprotein levels have continued for several decades (15). Since 2007, genome-wide association studies (GWAS) have implicated numerous common genetic variants in multiple loci and genes as influences in the determination of lipid and lipoprotein levels (16). Recently, a comprehensive meta-analysis of the GWAS performed a close linkage analysis between the *PABPC4* rs4660293 locus and plasma HDL-C concentrations (17). A further meta-analysis suggested that the *PABPC4* rs4660293 single nucleotide polymorphism (SNP) was correlated with C-reactive protein (CRP) levels (18), high levels of which are associated with increased risk of CHD mortality (19). Furthermore, Middelberg *et al* (20) reported that the *PABPC4* influenced plasma HDL between adolescents and adults. Poly(A) binding protein cytoplasmic 4 (inducible form; *PABPC4*) is expressed in numerous cell types and is a homolog of cytoplasmic poly(A) binding protein (PABPC), which predominantly mediates the effect of the poly(A) tail on translation (21,22). In addition to regulating translation initiation, PABPC controls the rate of mRNA deadenylation and is involved in mRNA decay (22). Teslovich *et al* (17) hypothesized that the SNP of rs4660293 was associated with HDL amongst populations of European ancestry. However, how the genetic associations described will apply to populations of diverse ancestry, particularly amongst Chinese people remains elusive.

There are 56 ethnic groups in China, of which the Han nationality is the largest group. The Mulao nationality is one of the 55 Chinese minorities, with a population of 207,352 according to the fifth national census statistics of China in 2000 (23). Ninety percent of the Mulao population reside in Luocheng Mulao Autonomous County, Guangxi Zhuang Autonomous region, China, where their history dates back to the Jin Dynasty (AD265–420) (24). A previous study indicated that the genetic association between individuals in the Mulao population and other minorities in Guangxi was markedly closer than that between the Mulao and Han or Uighur nationalities (25). The Mulao nationality has become a valuable subgroup for use in population genetic studies. However, to the best of our knowledge, there have been no previous studies performed to evaluate the association between the *PABPC4* rs4660293 SNP and serum lipid levels in this population. Therefore, the present study aimed to investigate the association between the *PABPC4* rs4660293 SNP and serum lipid levels amongst the Guangxi Mulao and Han populations.

## Materials and methods

### Study subjects

In the present study, 727 unrelated subjects of Mulao and 729 unrelated participants of Han Chinese heritage randomly selected from stratified randomized samples from a previous study by our group (26). All subjects were rural agricultural workers residing in Luocheng Mulao Autonomous County, Guangxi Zhuang Autonomous Region, China. The Mulao subjects comprised 329 (45.25%) males and 398 (54.75%) females, with a mean age of 52.92±15.24 years. The participants of Han nationality comprised 324 (44.44%) males and 405 (55.56%) females, with a mean age of 52.42±15.19 years. The total age range of subjects was 16–92 years. Subjects with diseases associated with atherosclerosis, CHD, diabetes or those who were using lipid-lowering medication (including statins, fibrates, beta-blockers, diuretics or hormones) were excluded from the study prior to blood sampling. The study design was approved by the Ethics Committee of the First Affiliated Hospital, Guangxi Medical University, Nanning, China. Informed consent was obtained from all subjects prior to their inclusion in the study.

### Epidemiological survey

An epidemiological survey was performed according to internationally standardized methods, following a common protocol (27). Information regarding demographic, socioeconomic status and lifestyle factors was collected via standardized questionnaires (27). The intake of alcohol was quantified as the number of liangs (~50 g) of rice wine, corn wine, rum, beer or liquor consumed during the preceding 12 months. Alcohol consumption was categorized into groups of ≤25 or >25 g alcohol per day. Smoking status was categorized into groups of ≤20 or >20 cigarettes per day. In the physical examination, several parameters, including height, weight and waist circumference, were measured. Sitting blood pressure was measured three times with a mercury sphygmomanometer following 5 min of rest and the average measurement was recorded. Systolic blood pressure was determined by the first Korotkoff sound and diastolic blood pressure by the fifth Korotkoff sound. Body weight, to the nearest 50 g, was measured using a portable balance scale. Subjects were weighed wearing minimal clothing with their shoes off. Height was measured, to the nearest 0.5 cm, using a stadiometer. Body mass index (BMI; kg/m^2^) was calculated using height and weight measurements.

### Biochemical measurements

Blood samples were obtained from subjects in a fasting state. Biochemical parameters, including total cholesterol (TC), triglyceride (TG), HDL-C and LDL-C, were measured using enzymatic methods with commercially available kits (Tcho-1 and TG-LH kits; Randox Laboratories Ltd., Crumlin, UK; Cholestest N HDL and Cholestest LDL; Daiichi Pure Chemicals Co., Ltd., Tokyo, Japan). Serum apolipoprotein (Apo) AI and ApoB concentrations were quantified by the immunoturbidimetric immunoassay using a commercial kit (APO CAL; cat. no. LP3023; Randox Laboratories, Ltd) (25). Fasting blood glucose was determined with a glucose meter (Accu-Chek; F. Hoffman-La Roche AG, Basel, Switzerland).

### DNA amplification and genotyping

Genomic DNA was isolated from peripheral blood leukocytes using the phenol-chloroform method (26). The extracted DNA was stored at 4°C prior to analysis. Genotyping of the *PABPC4* rs4660293 SNP was performed by polymerase chain reaction (PCR) and restriction fragment length polymorphism (RFLP) analyses. PCR amplification was performed using the following primers: Forward, 5′-CTCTGGGACCCTCTTCTT-3′ and reverse, 5′-CGTTTCACTTCGCTTTCT-3′ (Sangon Biotech Co., Ltd, Shanghai, China). Each amplification reaction was performed in a total volume of 25 *μ*l, containing 2 *μ*l genomic DNA, 1 *μ*l of each primer (10 pmol/l), 12.5 *μ*l 2x *Taq* PCR Mastermix (comprising 20 mM Tris-HCl, pH 8.3, 100 mM KCl, 3 mM MgCl_2_, 0.1 U *Taq* polymerase/*μ*l, 500 *μ*M of each deoxyribo-nucleotide; Sangon Biotech Co., Ltd) and 8.5 *μ*l double-distilled H_2_O (DNase/RNase-free). Processing began at 95°C for 5 min, followed by 45 sec of denaturing at 94°C, 45 sec of annealing at 52°C and 1 min of extension at 72°C for 30 cycles. The amplification was completed by a final extension at 72°C for 7 min. Subsequently, 10 U *Taq*I enzyme was added directly to the PCR products (10 *μ*l) and digested at 65°C overnight. Following restriction enzyme digestion of the amplified DNA, genotypes were identified by electrophoresis on 2% ethidium bromide-stained agarose gels and visualized using ultraviolet illumination (Universal Hood II; Bio-Rad Laboratories, Inc., Hercules, CA, USA). Genotypes were subsequently scored by an experienced reader blinded to the epidemiological and lipid results, as follows: Genotype AA=1, genotype AG=2, genotype GG=3.

### DNA sequencing

Three samples analyzed by PCR-RFLP were also evaluated by direct sequencing with an ABI Prism 3100 (Applied Biosystems Life Technologies, Foster City, CA, USA) at the Shanghai Sangon Biological Engineering Technology & Services Co., Ltd, Shanghai, China.

### Diagnostic criteria

The normal values of serum TC, TG, HDL-C, LDL-C, ApoAI, ApoB levels and the ratio of ApoAI to ApoB as designated by the Clinical Science Experiment Center (The First Affiliated Hospital, Guangxi Medical University) were 3.10–5.17, 0.56–1.70, 0.91–1.81 and 2.70–3.20 mmol/l as well as 1.00–1.78, 0.63–1.14 and 1.00–2.50 g/l, respectively (26). Hypertension was assessed according to the criteria outlined by the 1999 World Health Organization - International Society of Hypertension Guidelines for the management of hypertension (28). The categories of normal weight, overweight and obesity were defined as a BMI of <24, 24–28 and >28 kg/m^2^, respectively (28).

### Statistical analyses

Statistical analyses were performed using SPSS 16.0 (SPSS Inc., Chicago, IL, USA). Qualitative variables are expressed as raw counts and percentages. Quantitative variables are presented as the mean ± standard deviation, except serum TG levels, which are presented as medians and interquartile ranges. General characteristics between the Mulao and Han populations were compared by Student's unpaired t-test. Genotypic and allelic frequencies were calculated by direct counting and the standard goodness-of-fit test was used to investigate departures from the Hardy-Weinberg equilibrium. Differences in genotype distribution and gender ratio between the populations were determined by χ^2^ analysis. Analysis of covariance was used to evaluate the association between specific genotypes and serum lipid parameters. Factors that may influence serum lipid concentrations, including gender, age, BMI, blood pressure, alcohol consumption and cigarette smoking, were adjusted for statistical analysis. The association between serum lipid levels, genotypes and environmental factors was assessed by multiple linear regression analysis with stepwise modeling. Differences among the genotypes were determined using the Kruskal-Wallis test or the Wilcoxon-Mann-Whitney test. Two-tailed P<0.05 was considered to indicate a statistically significant difference between values.

## Results

### General characteristics and serum lipid levels

The comparison between general characteristics and serum lipid levels amongst the Mulao and Han populations are summarized in Table I. BMI, diastolic blood pressure and the ratio of ApoAI to ApoB were lower in the Mulao population than those in the Han population (P<0.05–0.01), whereas height, LDL-C and ApoB levels were higher in the Mulao population than those in the Han population (P<0.05–0.001). There were no significant differences in waist circumference, systolic blood pressure, pulse pressure, glucose, serum TC, TG, HDL-C, ApoAI, age distribution, gender ratio, the percentage of smokers or alcohol consumption between the two ethnic groups (P>0.05 for all).

### Results of electrophoresis and genotyping

**Figure 1:**
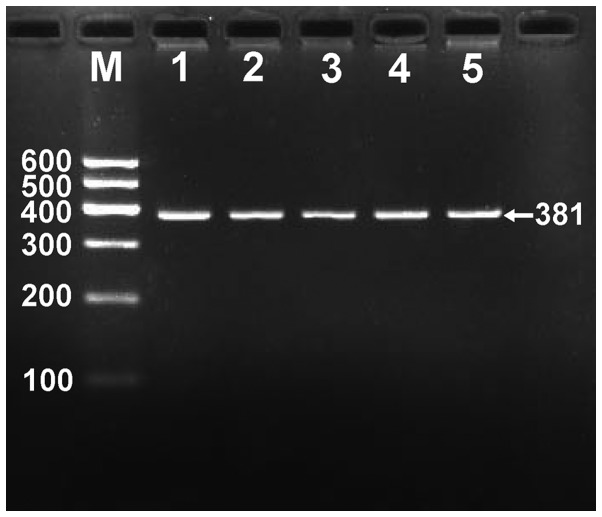
Electrophoresis of PCR products of the *PABPC4* rs4660293 single nucleotide polymorphism. Lanes: M, 100 bp marker ladder; 1–6, PCR products (381 bp). PCR, polymerase chain reaction.

**Figure 2:**
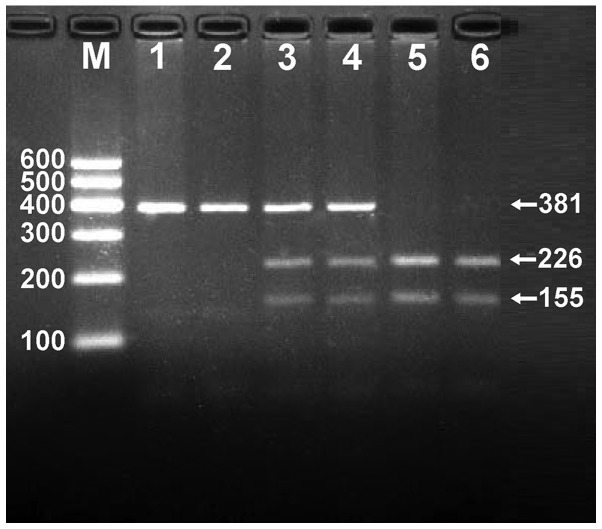
Genotyping of the *PABPC4* rs4660293 single nucleotide polymorphism. Lanes: M, 100 bp marker ladder; 1 and 2, AA genotype (381 bp); 3 and 4, AG genotype (381, 226 and 155 bp); 5 and 6, GG genotype (226 and 155 bp).

Genomic DNA collected from the sample populations was amplified by PCR and imaged by 2.0% agarose gel electrophoresis. The 381 bp nucleotide sequence gene of interest was detected in all samples (Fig. 1). The genotypes identified were named according to the presence or absence of enzyme restriction sites, with an A to G SNP at rs4660293. The presence of the cutting site indicated the G allele, while its absence indicated the A allele, which could not be cut. Therefore, the AA genotype was homozygous for the absence of the site (band at 381 bp), the AG genotype was heterozygous for the absence and presence of the site (bands at 381, 226 and 155 bp) and the GG genotype was homozygous for the presence of the site (bands at 226 and 155 bp; Fig. 2).

### Results of sequencing

**Figure 3:**
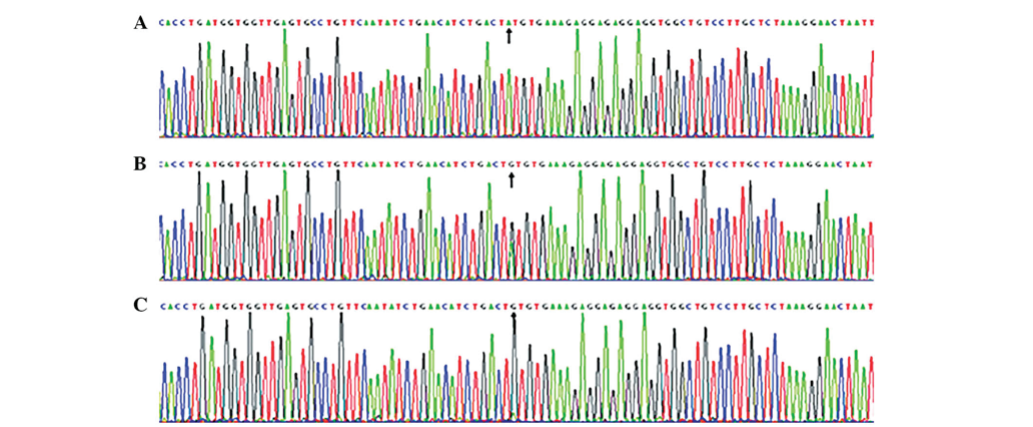
Part of the nucleotide sequence of the *PABPC4* rs4660293 single nucleotide polymorphism. (A) AA genotype; (B) AG genotype; (C) GG genotype.

The results were separated into AA, AG and GG genotypes of the rs4660293 SNP by PCR-RFLP and the genotypes were further confirmed by sequencing (Fig. 3).

### Genotypic and allelic frequencies

The genotypic and allelic distributions of the rs4660293 SNP are outlined in Table II. There was no significant difference in genotypic or allelic frequencies between the Mulao and Han populations. Differences in the genotypic and allelic frequencies were detected between Mulao males and females (P<0.05), but not between Han males and females. The frequency of the minor G allele in the Mulao population was higher in males (22.12%) than in females (13.44%, P=0.017).

### Genotypes and serum lipid levels

As depicted in Table III, the levels of LDL-C in the Han population varied between the AA and AG/GG genotypes (P<0.05). Following adjustments for age, gender, BMI, blood pressure, cigarette smoking and alcohol consumption, the G allele carriers were found to have higher LDL-C levels than the G allele non-carriers. Serum lipid parameters were analyzed according to gender and it was revealed that female, but not male G allele carriers in the Han population had higher TC, HDL-C and ApoAI levels, than G allele non-carriers (P<0.05 for each). Female, but not male G allele carriers in the Mulao population had lower TC and HDL-C levels than the G allele non-carriers (P<0.05 for each).

### Risk factors for serum lipid parameters

As described in Table IV, multiple linear regression analyses indicated that the levels of TC, HDL-C, ApoAI and ApoB in the Han population, but not the Mulao population, were correlated with the genotype (P<0.05). When the regression analysis was performed according to gender, it was revealed that the levels of TC, HDL-C and ApoAI in the Han population and TC and HDL-C in the Mulao population were associated with genotypes in females but not in males of the respective population (P<0.05–0.001).

Serum lipid parameters were also found to correlate with lifestyle factors, including gender, age, BMI, waist circumference, glucose, alcohol consumption, cigarette smoking and blood pressure amongst the two ethnic groups (P<0.05–0.001; Tables V and VI).

## Discussion

Disorders of lipid metabolism are associated with atherosclerotic cardiovascular disease. It has been demonstrated that lipid concentration is modulated by gene-lifestyle interactions (12–14). Numerous studies have indicated that ~40–60% of variation in serum lipid profiles was genetically determined (11), and that LDL-C, HDL-C and TG concentrations were influenced by the genetic constitution of a particular individual (13). The prevalence of dyslipidemia continues to increase worldwide, causing significant personal health problems as well as imposing a substantial economic burden on societies. Therefore, further study is required in order to increase the understanding of the risk factors underlying dyslipidemia in certain populations.

At present, the association between the *PABPC4* rs4660293 polymorphism and serum lipid levels remains to be elucidated. The genotypic and allelic frequencies of rs4660293 SNP in PABPC4 in diverse racial/ethnic groups are not well understood. The frequency of the G allele was revealed to be 23% amongst Europeans (17) and 25% amongst Australians (20). In the present study, it was demonstrated that there was no significant difference in the G allele frequency of the *PABPC4* rs4660293 SNP between the Mulao and Han populations examined (11.62 vs. 12.69%). Subgroup analyses revealed that there were no significant differences in genotypic and allelic frequencies between Han males or females, but that the minor allelic frequency was higher in Mulao males than that in Mulao females, and the genotypic distribution varied between Mulao females and males (P<0.05). These results suggested that the prevalence of the *PABPC4* rs4660293 SNP G allele may have racial/ethnic specificity, as well as gender specificity. Middelberg *et al* (20) reported that the *PABPC4* rs4660293 SNP was correlated with HDL-C in adults but not in adolescents. The results of the present study indicated that in the Han population, G allele carriers were associated with higher LDL-C levels and higher TC, HDL-C and ApoAI levels in females but not in males, than those of the G allele non-carriers. Of note, the Mulao female, but not male, G allele carriers had lower TC and HDL-C levels than those of the G allele non-carriers. These findings suggested that the association of *PABPC4* rs4660293 SNP and serum lipid levels varied between the Mulao and Han nationalities, and that there was a gender-specific association in the two ethnic groups.

Amongst individuals of the Mulao population, certain customs must be considered. The majority of the Mulao population resides in the Guangxi Zhuang Autonomous Region, China, which is characterized by an agricultural economy. The inhabitants of the region tend to share similar lifestyles, as well as eating habits, prefering to eat cold foods accompanied by acidic and spicy dishes, local bean soy sauce, pickled vegetables and animal offal, which contains a high proportion of saturated fatty acid (29). There is additionally a strict intra-ethnic marriage culture in Mulao society, where engagements are arranged by the family in childhood, with the female frequently being ~4–5 years older than the male. Furthermore, there is a tradition for marriage to an individual's mother's brother's daughter (30). Due to the conservative and isolated nature of the Mulao minority, the genetic background and certain lipid-associated genetic variants in this population were hypothesized to differ from those in the Han population (25). The above evidence may partly explain the discrepancies of the PABPC4 polymorphism and serum lipid levels observed between the Han and Mulao populations. Furthermore, environmental factors were positively correlated with serum lipid levels (11,31–33). In the present study, it was demonstrated that serum lipid parameters were associated with age, gender, alcohol consumption, cigarette smoking, BMI and blood pressure in the two ethnic groups. These results suggested that such environmental factors have a key role in determining the serum lipid levels in these study populations.

There are several potential limitations to the results obtained in the present study. The sample size may be not have been large enough to be representative of the populations as a whole. Despite the association between *PABPC4* rs4660293 SNP and serum lipid levels identified in the present study, numerous unmeasured genetic and environmental factors remain. Furthermore, gene-gene, gene-environment, and environment-environment interactions were not evaluated in the present study. Further studies comprising larger sample sizes and paying particular attention to gene-gene and gene-environment interactions, are required in order to confirm the results of the present study.

In conclusion, the present study demonstrated that the *PABPC4* rs4660293 SNP was associated with serum TC, HDL-C, LDL-C and ApoAI levels in the Mulao and Han populations, but that the genotypic and allelic frequencies of *PABPC4* rs4660293 SNP and the association of this SNP and serum lipid parameters varied between the two populations. A gender-specific association was also observed in the two ethnic groups. Ideal biomarkers may provide diagnostic information on patients at risk for dyslipidemic disease and/or prognostic information on patients with established dyslipidemic disease, they may also serve as predictors of efficacy for therapeutic interventions.

## Figures and Tables

**Table I:** Comparison of demographics, lifestyle characteristics and serum lipid levels between Mulao and Han populations.

Parameter	Han	Mulao	t (χ^2^)	P-value
Subjects (n)	729	727		
Male/Female	324/405	329/398	0.097	0.756
Age (years)	52.42±15.19	52.92±15.24	−0.622	0.534
Height (cm)	154.44±8.20	155.38±7.93	−2.221	0.026
Weight (kg)	53.65±9.06	52.90±9.41	1.543	0.123
Body mass index (kg/m^2^)	22.48±3.40	21.85±3.13	3.648	<0.000
Waist circumference (cm)	75.34±7.95	75.21±8.74	0.302	0.763
Cigarette smoking, n (%)				
Nonsmoker	515 (70.64)	538 (74.00)		
≤20 cigarettes/day	188 (25.79)	162 (22.28)	2.450	0.294
>20 cigarettes/day	26 (3.57)	27 (3.72)		
Alcohol consumption, n (%)				
Nondrinker	559 (76.68)	544 (74.79)		
≤25 g/day	77 (10.56)	64 (8.82)	4.589	0.101
>25 g/day	93 (12.76)	119 (16.39)		
Systolic blood pressure (mmHg)	130.27±19.36	129.43±22.00	0.779	0.436
Diastolic blood pressure (mmHg)	82.47±11.06	81.00±11.38	2.496	0.013
Pulse pressure (mmHg)	47.80±14.56	48.43±16.45	−0.763	0.445
Glucose (mmol/l)	6.04±1.60	6.01±1.64	0.294	0.769
Total cholesterol (mmol/l)	5.01±1.02	5.10±1.08	−1.795	0.073
Triglycerides (mmol/l)	1.07 (0.85)	1.07 (0.76)	−0.389	0.697
HDL-C (mmol/l)	1.74±0.54	1.78±0.45	−1.801	0.072
LDL-C (mmol/l)	2.87±0.82	3.01±0.86	−3.233	0.001
Apo AI (g/l)	1.34±0.26	1.34±0.40	0.138	0.890
Apo B (g/l)	0.85±0.20	1.01±0.59	−6.613	<0.000
Apo AI/Apo B	1.65±0.50	1.57±0.76	2.432	0.015

HDL-C, high-density lipoprotein cholesterol; LDL-C, low-density lipoprotein cholesterol; Apo, apolipoprotein.

**Table II:** Comparison of the genotype and allele frequencies of single nucleotide polymorphism in the Mulao and Han populations [n (%)].

Group	N	Genotype			Allele		
		AA	AG	GG	A		G
Han	729	555 (76.13)	163 (22.36)	11 (1.51)	1273 (87.31)		185 (12.69)
Mulao	727	566 (77.85)	153 (21.05)	8 (1.10)	1285 (88.38)		169 (11.62)
χ^2^			0.895			0.774	
P-value			0.639			0.379	
Han							
Male	324	250 (77.16)	66 (20.37)	8 (2.47)	566 (87.35)		82 (12.65)
Female	405	305 (75.31)	97 (23.95)	3 (0.74)	707 (87.28)		103 (12.72)
χ^2^			4.677			0.001	
P-value			0.096			0.972	
Mulao							
Male	329	268 (81.46)	60 (18.24)	1 (0.30)	596 (77.88)		62 (22.12)
Female	398	298 (74.87)	93 (23.37)	7 (1.76)	689 (86.56)		107 (13.44)
χ^2^			6.719			5.666	
P-value			0.035			0.017	

**Table III:** Comparison of the lipid and Apolipoprotein levels between genotypes of Mulao and Han population [n (%)].

Ethnic genotype	N	TC (mmol/l)	TG (mmol/l)	HDL-C (mmol/l)	LDL-C (mmol/l)	Apo AI (mmol/l)	Apo B (mmol/l)	ApoAI/ApoB
Han								
AA	555	4.97±1.05	1.08 (0.77)	1.71±0.43	2.83±0.82	1.34±0.27	0.85±0.20	1.65±0.51
AG/GG	174	5.11±0.92	0.98 (1.14)	1.81±0.78	2.98±0.80	1.36±0.25	0.87±0.19	1.65±0.47
F-value		−1.587	−0.888	−1.688	−2.073	−1.083	−1.152	0.135
P-value		0.113	0.375	0.093	0.038	0.279	0.255	0.893
Han/male								
AA	250	5.20±1.14	1.22 (0.89)	1.68±0.43	2.89±0.85	1.37±0.29	0.91±0.21	1.58±0.51
AG/GG	74	5.24±0.97	1.28 (1.25)	1.65±0.41	3.04±0.74	1.33±0.27	0.93±0.19	1.49±0.37
F-value		−0.282	−0.017	0.537	−1.427	0.826	−0.705	1.659
P-value		0.778	0.986	0.592	0.155	0.411	0.482	0.099
Han/female								
AA	305	4.79±0.94	1.00 (0.62)	1.74±0.42	2.79±0.80	1.31±0.25	0.80±0.18	1.72±0.50
AG/GG	100	5.02±0.88	0.87 (0.86)	1.94±0.96	2.94±0.85	1.38±0.23	0.83±0.18	1.76±0.50
F-value		−2.193	−0.969	−2.055	−1.553	−2.449	−1.214	−0.857
P-value		0. 029	0.333	0.042	0.121	0.015	0.226	0.392
Mulao								
AA	566	5.14±1.04	1.07 (0.77)	1.79±0.45	3.03±0.85	1.35±0.39	1.02±0.60	1.56±0.68
AG/GG	161	4.98±1.18	1.07 (0.68)	1.75±0.47	2.95±0.90	1.31±0.42	0.97±0.54	1.60±0.97
F-value		1.632	−0.955	0.981	1.029	1.026	0.861	−0.668
P-value		0.103	0.344	0.327	0.304	0.305	0.399	0.505
Mulao/male								
AA	268	5.05±1.09	1.11 (0.96)	1.74±0.51	2.93±0.83	1.34±0.39	1.06±0.69	1.54±0.68
AG/GG	61	5.08±1.10	1.10 (0.99)	1.76±0.53	2.91±0.86	1.30±0.50	0.96±0.48	1.54±0.82
F-value		−0.242	−0.12	−0.36	0.177	0.666	1.026	−0.024
P-value		0.809	0.904	0.719	0.866	0.506	0.305	0.981
Mulao/female								
AA	298	5.22±0.99	1.04 (0.64)	1.84±0.37	3.12±0.85	1.35±0.39	0.98±0.49	1.58±0.69
AG/GG	100	4.92±1.23	1.01 (0.60)	1.74±0.43	2.98±0.92	1.31±0.36	0.98±0.58	1.64±1.05
F-value		2.466	−0.98	2.12	1.411	0.803	0.023	−0.701
P-value		0.014	0.327	0.035	0.159	0.422	0.982	0.483

TC, total cholesterol; TG, triglycerides; HDL-C, high-density lipoprotein cholesterol; LDL-C, low-density lipoprotein cholesterol; Apo, apolipoprotein; Apo AI/Apo B, ratio of Apo AI to Apo B. Differences among the genotypes were determined using the Kruskal-Wallis test or the Wilcoxon-Mann-Whitney test. Values are expressed as the mean ± standard deviation, except serum TG levels, which are presented as medians and interquartile ranges.

**Table IV:** Correlation between serum lipid parameters and genotypes in Mulao and Han populations.

Lipid parameter	Genotype	Unstandardized coefficient	Std. error	Standardized coefficient	t	P-value
Han						
TC	Genotype	0.218	0.081	0.099	2.696	0.007
HDL-C	Genotype	0.129	0.048	0.103	2.667	0.008
ApoAI	Genotype	0.050	0.022	0.083	2.301	0.022
ApoB	Genotype	0.032	0.015	0.072	2.171	0.030
Han/female						
TC	Genotype	0.229	0.101	0.107	2.271	0.024
HDL-C	Genotype	0.237	0.072	0.167	3.292	0.001
ApoAI	Genotype	0.091	0.028	0.162	3.239	0.001
Mulao/female						
TC	Genotype	−0.300	0.119	−0.122	−2.528	0.012
HDL-C	Genotype	−0.097	0.043	−0.108	−2.251	0.025

TC, total cholesterol; HDL-C, high-density lipoprotein cholesterol; LDL-C, low-density lipoprotein cholesterol; Apo, apolipoprotein; std., standard.

**Table V:** Correlation between the lipid parameters and relative factors in Mulao and Han populations.

Lipid parameter	Risk factor	Unstandardized coefficient	Std. error	Standardized Coefficient	t	P-value
Han and Mulao						
TC	Waist circumference	0.014	0.004	0.114	3.188	0.001
	Age	0.012	0.002	0.175	6.709	<0.001
	Body mass index	0.036	0.011	0.116	3.229	0.001
	Ethnic group	0.155	0.054	0.076	2.879	0.004
TG	Waist circumference	0.040	0.005	0.258	7.407	<0.001
	Alcohol consumption	0.005	0.001	0.140	5.530	<0.001
	Glucose	0.093	0.020	0.116	4.568	<0.001
	Body mass index	0.028	0.013	0.071	2.058	0.040
HDL-C	Waist circumference	−0.008	0.002	−0.136	−3.674	<0.001
	Alcohol consumption	0.001	0.000	0.119	4.011	<0.001
	Gender	0.112	0.030	0.113	3.715	<0.001
	Age	0.002	0.001	0.055	2.101	0.036
	Body mass index	−0.015	0.005	−0.099	−2.764	0.006
LDL-C	Body mass index	0.037	0.009	0.147	4.109	<0.001
	Age	0.010	0.001	0.173	6.649	<0.001
	Ethnic group	0.161	0.045	0.096	3.623	<0.001
	Alcohol consumption	−0.002	0.001	−0.072	−2.724	0.007
	Waist circumference	0.009	0.004	0.094	2.620	0.009
ApoAI	Alcohol consumption	0.002	0.000	0.216	7.214	<0.001
	Waist circumference	−0.004	0.001	−0.098	−3.560	<0.001
	Gender	0.048	0.021	0.071	2.331	0.020
Apo B	Waist circumference	0.011	0.001	0.208	7.975	<0.001
	Ethnic group	0.158	0.023	0.176	6.826	<0.001
	Glucose	0.033	0.007	0.119	4.548	<0.001
	Pulse pressure	0.002	0.001	0.061	2.322	0.020
ApoAI/Apo B	Waist circumference	−0.014	0.003	−0.183	−5.012	<0.001
	Glucose	−0.041	0.010	−0.103	−3.969	<0.001
	Alcohol consumption	0.002	0.000	0.131	4.520	<0.001
	Body mass index	−0.021	0.007	−0.112	−3.152	0.002
	Gender	0.118	0.038	0.092	3.105	0.002
Han						
TC	Waist circumference	0.021	0.006	0.178	3.662	<0.001
	Age	0.015	0.002	0.232	6.345	<0.001
	Alcohol consumption	0.003	0.001	0.117	3.168	0.002
	Body mass index	0.029	0.013	0.107	2.223	0.027
TG	Waist circumference	0.057	0.006	0.331	9.441	<0.001
	Alcohol consumption	0.007	0.001	0.174	4.620	<0.001
	Glucose	0.147	0.030	0.171	4.913	<0.001
	Cigarette smoking	0.018	0.006	0.115	3.049	0.002
HDL-C	Waist circumference	−0.012	0.003	−0.179	−4.647	<0.001
LDL-C	Waist circumference	0.015	0.005	0.143	2.909	0.004
	Age	0.011	0.002	0.208	5.477	<0.001
	Body mass index	0.031	0.012	0.133	2.718	0.007
	Glucose	0.050	0.020	0.097	2.524	0.012
ApoAI	Alcohol consumption	0.003	0.000	0.356	8.793	<0.001
	Body mass index	−0.010	0.003	−0.133	−3.624	<0.001
	Gender	0.092	0.024	0.178	3.863	<0.001
	Cigarette smoking	0.005	0.001	0.167	3.723	<0.001
	Age	0.001	0.001	0.087	2.407	0.016
Apo B	Waist circumference	0.006	0.001	0.267	5.849	<0.001
	Glucose	0.021	0.004	0.177	5.226	<0.001
	Body mass index	0.011	0.002	0.196	4.412	<0.001
	Gender	−0.050	0.014	−0.129	−3.649	<0.001
	Pulse pressure	0.001	0.000	0.077	2.235	0.026
ApoAI/Apo B	Waist circumference	−0.012	0.003	−0.198	−4.187	<0.001
	Alcohol consumption	0.003	0.001	0.216	5.588	<0.001
	Gender	0.242	0.043	0.252	5.582	<0.001
	Body mass index	−0.029	0.006	−0.212	−4.642	<0.001
	Cigarette smoking	0.008	0.002	0.151	3.494	0.001
	Glucose	−0.031	0.010	−0.104	−2.979	0.003
Mulao						
TC	Age	0.010	0.003	0.140	3.806	<0.001
	Body mass index	0.057	0.013	0.167	4.526	<0.001
TG	Waist circumference	0.040	0.005	0.287	7.896	<0.001
	Alcohol consumption	0.002	0.001	0.082	2.259	0.024
HDL-C	Body mass index	−0.027	0.007	−0.186	−3.568	<0.001
	Waist circumference	−0.006	0.003	−0.115	−2.217	0.027
LDL-C	Body mass index	0.053	0.010	0.194	5.285	<0.001
	Age	0.007	0.002	0.124	3.370	0.001
	Gender	0.169	0.064	0.097	2.646	0.008
ApoAI	Alcohol consumption	0.001	0.000	0.111	2.943	0.003
	Waist circumference	−0.005	0.002	−0.103	−2.716	0.007
Apo B	Waist circumference	0.012	0.002	0.182	4.905	<0.001
	Glucose	0.048	0.013	0.135	3.651	<0.001
ApoAI/B	Waist circumference	−0.019	0.003	−0.220	−5.946	<0.001
	Glucose	−0.048	0.017	−0.103	−2.798	0.005

TC, total cholesterol; TG, triglycerides; HDL-C, high-density lipoprotein cholesterol; LDL-C, low-density lipoprotein cholesterol; Apo, apolipoprotein; std., standard.

**Table VI:** Correlation between the lipid parameters and relative factors in males and females of Mulao and Han populations.

Lipid parameter	Risk factor	Unstandardized coefficient	Std. error	Standardized coefficient	t	P-value
Han/male						
TC	Waist circumference	0.024	0.007	0.212	3.580	<0.001
TG	Waist circumference	0.074	0.011	0.351	6.501	<0.001
	Alcohol consumption	0.008	0.002	0.208	3.800	<0.001
	Glucose	0.157	0.054	0.159	2.915	0.004
	Cigarette smoking	0.020	0.009	0.123	2.219	0.027
HDL-C	Waist circumference	−0.012	0.003	−0.250	−4.348	<0.001
	Alcohol consumption	0.001	0.000	0.156	2.671	0.008
	Cigarette smoking	0.005	0.002	0.132	2.256	0.025
LDL-C	Body mass index	0.039	0.012	0.193	3.295	0.001
	Cigarette smoking	0.014	0.004	−0.182	−3.103	0.002
ApoAI	Alcohol consumption	0.003	0.000	0.448	8.507	<0.001
	Cigarette smoking	0.005	0.001	0.185	3.522	0.001
	Waist circumference	−0.004	0.002	−0.126	−2.432	0.016
Apo B	Waist circumference	0.006	0.002	0.251	3.811	<0.001
	Body mass index	0.013	0.003	0.269	4.095	<0.001
	Glucose	0.015	0.006	0.139	2.607	0.010
ApoAI/ApoB	Body mass index	−0.029	0.007	−0.245	−3.899	<0.001
	Alcohol consumption	0.003	0.001	0.281	5.425	<0.001
	Cigarette smoking	0.009	0.002	0.208	4.012	<0.001
	Waist circumference	−0.011	0.004	−0.199	−3.173	0.002
Han/female						
TC	Age	0.020	0.003	0.307	6.256	<0.001
	Waist circumference	0.024	0.006	0.187	3.924	<0.001
	Glucose	0.062	0.031	0.097	1.996	0.047
TG	Waist circumference	0.041	0.006	0.307	6.406	<0.001
	Glucose	0.139	0.031	0.213	4.443	<0.001
	Waist circumference	−0.009	0.004	−0.104	−2.041	0.042
LDL-C	Age	0.018	0.003	0.315	6.655	<0.001
	Waist circumference	0.028	0.005	0.246	5.190	<0.001
	Alcohol consumption	0.003	0.001	0.135	2.649	0.008
	Body mass index	−0.012	0.004	−0.140	−2.779	0.006
	Age	0.002	0.001	0.101	1.993	0.047
Apo B	Waist circumference	0.006	0.002	0.252	3.894	<0.001
	Glucose	0.023	0.006	0.191	4.056	<0.001
	Pulse pressure	0.002	0.001	0.125	2.533	0.012
	Age	0.001	0.001	0.112	2.237	0.026
	Body mass index	0.008	0.004	0.127	1.978	0.049
ApoAI/ApoB	Waist circumference	−0.021	0.003	−0.319	−6.585	<0.001
	Pulse pressure	−0.006	0.002	−0.158	−3.264	0.001
Mulao/male						
TC	Body mass index	0.059	0.020	0.169	2.994	0.003
TG	Waist circumference	0.027	0.012	0.178	2.279	0.023
	Body mass index	0.091	0.034	0.207	2.678	0.008
	Glucose	0.109	0.041	0.144	2.644	0.009
HDL-C	Body mass index	−0.044	0.009	−0.270	−4.908	<0.001
	Alcohol consumption	0.001	0.000	0.159	2.891	0.004
LDL-C	Body mass index	0.039	0.015	0.147	2.583	0.010
Apo AI	Alcohol consumption	0.001	0.000	0.180	3.192	0.002
Apo B	Waist circumference	0.010	0.004	0.135	2.369	0.018
	Pulse pressure	0.005	0.002	0.125	2.193	0.029
ApoAI/Apo B	Waist circumference	−0.019	0.004	−0.251	−4.531	<0.001
	Alcohol consumption	0.001	0.001	0.110	1.977	0.049
Mulao/female						
TC	Age	0.013	0.003	0.188	3.885	<0.001
	Body mass index	0.058	0.017	0.169	3.478	0.001
TG	Waist circumference	0.027	0.007	0.205	4.156	<0.001
HDL-C	Body mass index	−0.035	0.006	−0.279	−5.787	<0.001
LDL-C	Body mass index	0.064	0.013	0.228	4.751	<0.001
	Age	0.012	0.003	0.206	4.290	<0.001
Apo B	Waist circumference	0.014	0.003	0.214	4.449	<0.001
	Glucose	0.072	0.017	0.208	4.312	<0.001
ApoAI/Apo B	Waist circumference	−0.021	0.005	−0.210	−4.294	<0.001
	Age	−0.007	0.003	−0.139	−2.844	0.005

TC, total cholesterol; TG, triglycerides; HDL-C, high-density lipoprotein cholesterol; LDL-C, low-density lipoprotein cholesterol; Apo, apolipoprotein; std., standard.
